# Microcapsule Techniques to Emphasize Functional Plant Oil Quality and Their Applications in the Food Industry: A Review

**DOI:** 10.3390/foods14040677

**Published:** 2025-02-17

**Authors:** Zhiran Zhang, Fei Li, Ziyan Zhang, Atif Muhmood, Shengxin Li, Mengkai Liu, Sen Zhou, Zubo Du, Chongchong Ruan, Jie Sun

**Affiliations:** 1 College of Life Sciences, Qingdao University, Qingdao 266071, China; zzrzgj@163.com (Z.Z.); pdlifei@163.com (F.L.); zhangziyan77@126.com (Z.Z.); lsxy0215@163.com (S.L.); liumengkaikai@163.com (M.L.); jason_zhousen@163.com (S.Z.); 2 Shandong Luhua Group Co., Ltd., Laiyang 265200, China; gyldzb@luhua.cn; 3 Department of Agroecology, Aarhus University, 8000 Aarhus, Denmark; atif@agro.au.dk; 4 Guangdong Chubang Food Co., Ltd., Yangjiang 529500, China; ruanchongchong@mwx.cn

**Keywords:** microencapsulation, functional plant oils, food industry applications

## Abstract

Natural functional plant oils (FPOs) have been widely exploited due to their abundant biological activities. However, when exposed to oxygen, light, moisture, and heat, some limitations such as oxidative deterioration, impaired flavor, loss of nutritional value and volatile compounds, and decreased shelf life hinder the widespread application of FPOs in the food industry. Notably, the microencapsulation technique is one of the advanced technologies, which has been used to maintain the biological and physicochemical properties of FPOs. The present review provided a comprehensive overview of the nutrient compositions and functionality of FPOs, preparation techniques for microcapsules, and applications of microencapsulated FPOs (MFPOs) in the food industry. FPOs obtained from a wide range of sources were abundant in bioactive compounds and possessed disease risk mitigation and improved human health properties. The preparation methods of microencapsulation technology included physical, chemical, and physicochemical methods, which had the ability to enhance oxidative stability, functional, shelf life, and thermostability properties of FPOs. In this context, MFPOs had been applied as a fortification in sausage, meat, bakery, and flour products. Overall, this work will provide information for academic fields and industries the further exploration of food and nutriment products.

## 1. Introduction

Many functional ingredients naturally sourced from plant oils have shown to benefit our health and gained an increasing popularity and scientific interest in the food industry [1]. They are abundant in vital unsaturated fatty acids as well as bioactive compounds (vitamins, squalene, etc.) and possessed preventive and therapeutic effects on various deficiencies and endogenous diseases, including hypertension, heart disease, cancer, diabetes, etc. [1]. Adding functional ingredients to the food system and maintaining their nutritional properties and positive qualities are challenges during food processing and handling. These FPOs are prone to oxidative deterioration in food and nutraceutical formulations, which leads to negative quality and nutritional issues such as an off-flavor, nutrient loss, low physical stability and bio-accessibility, and reduction in shelf life [2].

Encapsulation, a process of building a functional barrier between the core and the wall material, is one of the most effective and innovative manners to avoid chemical and physical reactions and to maintain the functional and physicochemical properties of the core materials [3]. Commonly used materials include proteins, polysaccharides, and liposomes. Among them, the different hydrophilic and lipophilic groups make them play a key role as emulsifiers in the emulsion liquid system, reducing the interfacial tension between the oil phase and the water phase, forming a stable water-in-oil or oil-in-water system, and the polysaccharide is non-toxic, biodegradable, renewable, easy to modify, and a source of renewable raw materials [3]. In addition, they are inexpensive and readily available, have good solubility, and can maintain low viscosity even with high solid content [4]. Liposomes are spherical vesicle structures composed of phospholipids, in which a lipophilic bilayer sandwiched between two hydrophilic layers can encapsulate hydrophilic and hydrophobic drugs, which can prevent the contact between the encapsulated components and harmful foreign substances, protect subtle bioactive elements, and improve their effectiveness [3]. This technology for structuring FPOs could improve the overall acceptability and functional characteristics of the food product [4,5]. Microencapsulation is a typical technique in which the sensitive oils are surrounded by the external wall materials, which contributes to protect FPOs from oxidation triggered by heat, oxygen, light, metal ions, and moisture and to improve stability and bioavailability during processing, storage, and transportation [6,7].

**Figure 1:**
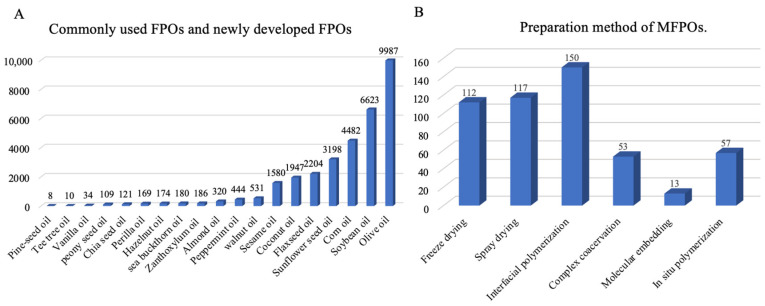
The number of studies from 2010 to 2025. (**A**) Commonly used FPOs and newly developed FPOs. (**B**) Preparation method of MFPOs.

**Figure 2:**
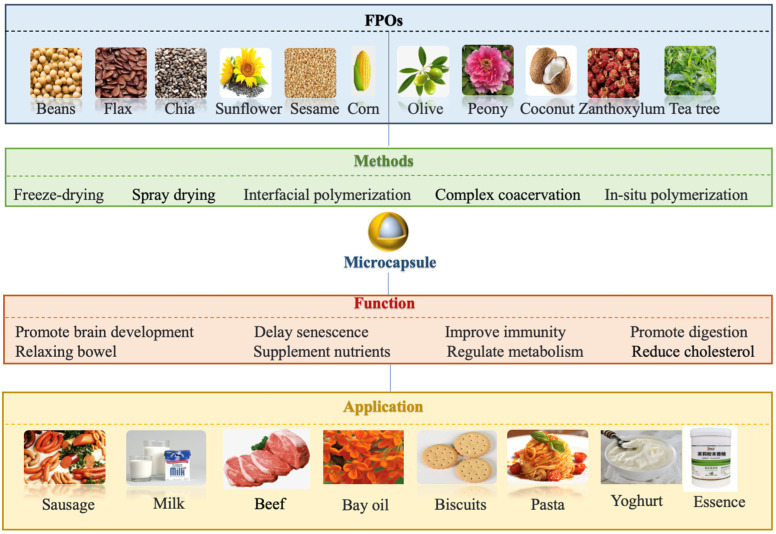
Sources, nutritional compositions, and applications of FPOs.

Pattnaik and Mishra have reviewed the blending and microencapsulation of bioactive enriched vegetable oil [8]. Moreover, Timilsena et al. have described a comprehensive overview of the relevant scientific literature on complex coacervation in microencapsulation of polyunsaturated fatty acid (PUFA)-rich plant oils [9]. Therefore, we summarized the most studied vegetable oils and their encapsulation strategies in various countries from 2010 to 2025 (Figure 1A,B), and we found that a comprehensive overview of the source, nutrition, composition, and biological characteristics of individual FPOs, as well as the in-depth application of microencapsulation methods in the food industry, is lacking, and this paper fills this gap (Figure 2). Furthermore, the challenges and prospects of FPOs utilizing microcapsule technology are discussed. Overall, this review provides valuable references for the academic field and industrial applications of FPOs.

## 2. Nutrient Compositions and Functional Activities of FPOs

FPOs have attracted the interest of researchers and widespread in the fields of condiments, cosmetics, and anticorrosive material due to their multiple nutrients and excellent bioactivities, such as hypertension and hyperlipidemia [10]. Therefore, a comprehensive summary outlining the specific nutritional compositions and bioactive compounds of FPOs derived from both herbaceous and woody sources is presented here.

### 2.1. FPOs Derived from Herbaceous Plants

In comparation of woody plants, FPOs derived from herbaceous plants possess more exceptional resilience to extreme environmental conditions and extensive and rapid cultivation. Moreover, herbaceous plants offer numerous advantages, such as cost-effectiveness, short growth cycle, and their suitability for large-scale cultivation [11]. The notable functional compositions and health benefits of FPOs (flax seed oil, chia seed oil, soybean oil, corn oil, sesame oil, sunflower oil, sea buckthorn oil, almond oil, perilla seed oil, peppermint oil, and vanilla oil) are exhibited in Table 1.

Alpha-linolenic acid (ALA), belonging to omega-3 PUFAs, is present in certain herb plant oils. Flaxseed contained 35–45% oil, with ALA accounting for more than half of its total fatty acids [12]. Similarly, perilla seeds with 17–42.7% oil contents rich in ALA (47–64% of total oil content) were valuable resources for both food and natural medicine [13]. Chia seeds, which are commonly used as nutritional supplements in various foods, contain a high amount of ALA, accounting for 64.39% of their total oil content [14]. Likewise, corn oil, sesame oil, and sunflower oil have been found to contain over 60% unsaturated fatty acids [15,16,17,18].

Omega-3 PUFAs can mitigate the risk of disease, including the adjustment of endothelial function, the reduction in plasma triglyceride levels, anti-inflammatory, and antithrombotic [19]. The protective effect of ALA against cardiovascular disease was particularly obvious when sufficient omega-3 fatty acids were consumed throughout the diet, which was associated with down-regulating pro-inflammatory and pro-atherogenic genes [20]. Furthermore, ALA-rich oil has been shown to reduce oxidative stress and CD40 ligand levels [21]. ALA can protect primary rat hepatocytes from stearic acid lipotoxicity-induced endoplasmic reticulum stress-mediated apoptosis and also safeguard renal cells against palmitic acid lipotoxicity by inhibiting endoplasmic reticulum stress [22]. Soybean oil, which contains 22–30% oleic acid (OA), is considered the most commonly used cooking oil worldwide [23]. Incorporating soya oil into food products has been shown to effectively reduce atherosclerosis and thrombogenic index [24].

In addition to being rich in unsaturated fatty acids, herbal FPOs also contain a significant amount of phytosterols, beta-carotene, polyphenols (tocopherol, carvones, limonene, etc.), which have various bioactivities, such as anti-inflammatory, antidiabetic, and anticancer effects [25]. Among these components, tocopherol plays a particularly important role. As shown in Table 1, the tocopherol content of herbal FPOs ranged from 69.8 to 790.2 mg/kg, which can contribute to the prevention and treatment of cancer, immune dysregulation, and neurological complications, as well as providing antioxidant and radioprotective capabilities [18,25]. Moreover, compounds such as carvone and limonene can destroy cell membranes, resulting in changes in electron flow, proton driving force, and active transport, as well as coagulation of cell contents, which effectively inhibit the growth of microorganisms such as *E. coli*, etc. [26]. Phytosterols could increase the content of low-density lipoprotein, and beta-carotenes have certain antioxidant properties, both of which have good preventive effects on cardiovascular disease [27]. Therefore, FPOs can be added as a dietary supplement to common cooking oils to improve their low nutritional characteristics.

### 2.2. FPOs Derived from Woody Plants

Currently, the industry of woody FPOs is being promoted by numerous countries worldwide. Although woody plants have a longer growth cycle, they require lower amounts of fertilizers and pesticides compared to herbaceous plants. Camellia (*Elaeis guineensis Jacq*), olive (*Olea europaea*), tea oil tree (*Camellia oleifera Abel*), and coconut palm (*Cocos nucifera* L.) are known as the four largest woody oil plants in the world [28].

Similarly, the FPOs derived from woody plants possess significant health benefits due to their high content of unsaturated fatty acids (Table 1). Olive oil, a crucial component in the Mediterranean diet, is particularly noteworthy due to its extremely high oleic acid content (56–84%). Olive oils have been reported to prevent cytokine-induced oxidative damage, maintain glucose levels and carbohydrate absorption, and improve insulin sensitivity [29]. The tea tree oil (TTO), known as “Oriental olive oil”, exhibited hepatoprotective effects against CCl_4_-induced oxidative damage due to its high unsaturated fatty acid content (90%) [30]. Furthermore, diet supplementation with coconut oil riche in lauric acid (45–50%) can decrease weight loss and cholesterol levels while improving insulin sensitivity [31].

In addition to being rich in unsaturated fatty acids, FPOs obtained from woody plants also contained abundant functional components, including squalene, flavonoids, and vitamin E. Among them, squalene can increase the stability of the oil and extend its shelf life, and it also has antioxidant, anticancer, anti-inflammatory, and anti-atherosclerosis functions [32]. As reported, squalene inhibited the oxidation of arachidonic acid and docosahexaenoic acid by 50% and LA by 22%, respectively [33]. Similar observations for walnut oil were reported by Zhang et al. In detail, after adding walnut oil for 20 days, catechin, quercetin, and caffeic acid reduced the aminoglycoside value (PAV) by 37.28%, 37.58%, and 36.26%, respectively, compared with the control group, indicating that walnut oil had significant antioxidant effect [34]. All in all, FPOs extracted from woody plants offered notable and extensive nutritional benefits.

**Table 1:** Nutritional composition and edible value of common FPOs.

Classification		Name	Saturated Fatty Acid	Monounsaturated Fatty Acids	Polyunsaturated Fatty Acids	Other Active Ingredients	Oil Content	Unsaturated Fatty Acid Content	Potential Health Benefits	Reference
FPOs from herbal plants		flaxseed oil	stearic acid, palmitic acid	OA (15–20%)	ALA (50–55%)	lignans, fiber, phytosterols, vitamin E, β-carotene, flavonoid, polyphenols	>35%	>75%	reduction in cardiovascular disease, atherosclerosis, diabetes, cancer, arthritis, osteoporosis, and autoimmune and neurological disorders	[12,35]
		chia seed oil	palmitic acid, stearic acid	OA (8.61%)	ALA (65. 91%) LA (18.66%)	vitamin E, squalene, carotenoid, phytosterol, polyphenols	>25%	>80%	supplement nutrition, Improve memory, increase satiety, reduce blood sugar	[36,37]
		soybean oil	palmitic acid	OA (22–30%)	LA (50–60%) ALA (7%)	tocopherols, phytosterols, soy isoflavones, vitamin E, phytosterol, vitamin D	>16%	>80%	Prevention of cardiovascular disease, delaying senescence, Metastasis boosting, promote brain development, Prevent vision loss	[38,39]
		corn oil	palmitic acid, stearic acid	OA (11.84%)	LA (57.74%) ALA (1%)	phytosterol, squalene, lecithin, vitamin E	>5%	>80%	reduce cholesterol, delaying senescence, Prevention of cardiovascular disease	[38,40]
		sesame oil	stearic acid, palmitic acid, arachidic acid	OA (38.84%)	LA (45.23%) ALA (0.61%)	phytosterol, vitamin E, sesame lignans	>35%	>85%	improve anemia, relieve a cough, relaxing bowel, delaying senescence, promote blood circulation	[41,42]
		sunflower seed oil	palmitic acid, stearic acid	OA (19.81%)	LA (64.35%) ALA (1%)	vitamin E, vitamin B, flavonoid, folate, niacin	>40%	>90%	delaying senescence, cure for insomnia, improve memory, reduce blood press, prevention of cancer	[38,43,44]
		sea buckthorn seed oil	palmitic acid, stearic acid, myristic acid	OA (17.8–23.8%)	LA (33.6–37.8%) ALA (24.1–29.3%)	flavone, carotene, phytosterol, vitamin E	>10%	>80%	cardiovascular protection, delaying senescence, blood lipid reduction, liver protection, vision protection	[45,46]
		almond oil	palmitic acid, palmitoleic acid, stearic acid	OA (67.3%)	LA (19.4%)	vitamin E, amygdalin	>40%	>90%	moistening lungs, tonifying spleen, anticancer, delaying senescence	[47]
		perilla oil	palmitic acid, stearic acid	OA (9–20%)	ALA (47–64%) LA (10–24%)	vitamin E, flavone, coumarin lactone	>15%	>90%	antiallergic, antimicrobials, anticancer drugs, attenuates immunoglobulin a nephropathy, prevents excessive growth of visceral adipose tissue	[13]
		peppermint oils	ND	ND	ND	carvones, menthone, alkaloid, menthol	<5.4%	ND	antioxidant, antibacterial	[48]
		vanilla oil	ND	ND	ND	vanillin, tannins, polyphenols	<5.07%	ND	antioxidant, antimutagenic and hypolipidemic activity	[49,50]
FPOs from woody plants	trees	olive oil	palmitic acid, stearic acid	OA (68.94%)	LA (12.22%) ALA (0.79%)	phytosterol, squalene	>20%	>80%	prevention of diabetes, anticancer, heart protection, immune regulation, anti-proliferation, anti-oxidation	[51,52]
		coconut oil	palmitic acid, lauric acid, stearic acid, myristic acid	OA (6.49%)	LA (1.91%)	vitamin E, polyphenol	>20%	>5%	anti-oxidation, anti-polymerization	[53,54]
		walnut oil	palmitic acid, stearic acid	OA (13.4%),	LA (55.3%) ALA (10%)	squalene, flavone, polyphenol, vitamin E	>60%	>90%	boost immunity, regulate cholesterol levels, promote brain and nervous system development, relaxing bowel, sleep improvement, promote bone growth,	[38,47]
		pine-seed oil	palmitic acid stearic acid	OA (14.6–48.5%)	LA (35.2–58.2%) pinolenic acid (0.2–22.4%)	vitamin A, vitamin E, phytosterol	>70%	>90%	delaying senescence, moistening of the intestine, nourishing the brain	[55,56]
	shrubs	hazelnut oil	palmitic acid, stearicacid	OA (80.48%),	LA (12.19%) ALA (0.05%) arachidic acid (0.08%)	vitamin A, vitamin E, squalene, β-sitosterol	>50%	>90%	prevent atherosclerosis, anti-aging, improve immunity, promote cholesterol degradation	[47,57]
		zanthoxylum oil	palmitic acid	OA (7%)	LA (18%) ALA (15%)	amide, alkaloid	>15%	>90%	supplement nutrition, regulate blood lipids, improve immunity, antiallergic	[58,59]
		TTO	palmitic acid stearic acid	OA (16.63–18.84%)	LA (24.51–25.16%) ALA (29.57–31.69%)	phytosterol, squalene, vitamin E, polyphenols	>25%	>90%	lower triglycerides and cholesterol, thereby preventing high blood pressure, heart disease, arteriosclerosis and other diseases	[60,61]
		peony seed oil	palmitic acid, stearic acid	OA (22%)	LA (27%) ALA (45%)	squalene, vitamin E, phytosterol, polyphenol	>25%	>90%	relaxing bowel, cure for insomnia, reduce blood press, reduce blood lipid	[62]

ND: not detected. TTO: tea tree oil, OA: oleic acid, ALA: alpha-linolenic acid, LA: linoleic acid.

## 3. The Preparation Process of FPO Microcapsules

The utilization of microcapsule tools can enhance FPOs’ oxidative stability and preserve their flavor, thereby serving as an effective protective barrier [63]. Depending on the material and preparation process, microencapsulation methods for edible bioactive substances can be categorized as follows: (a) physical methods (spray drying and freeze drying); (b) chemical methods (interfacial polymerization); (c) physical–chemical methods (complex coacervation, ionic gelation, and molecular embedding) [64] (Figure 2).

### 3.1. Physical Methods

#### 3.1.1. Freeze Drying

**Figure 3:**
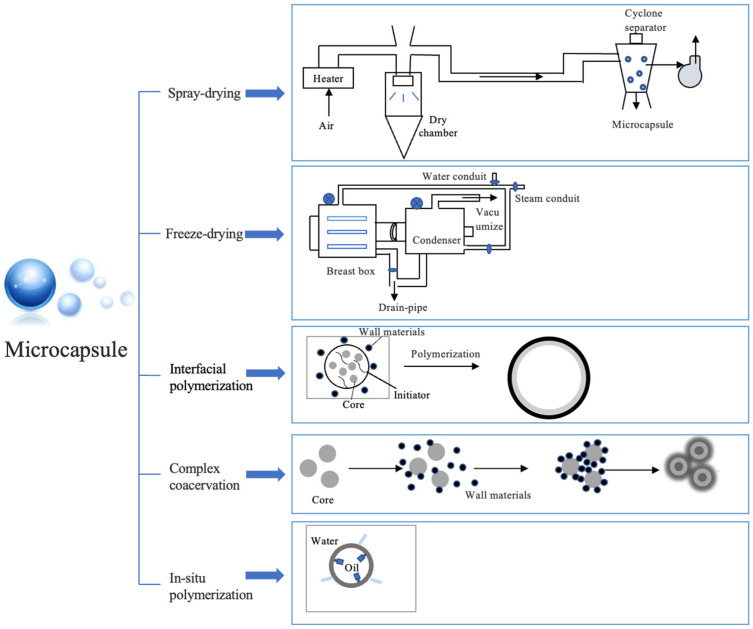
Methods for microcapsule preparation.

Freeze drying involves FPOs into the wall material, followed by freezing, ice sublimation, and desorption [65]. This method enables the conversion of microcapsules from liquid to solid under low temperature and vacuum conditions (Figure 3). The freeze drying process can effectively regulate the moisture content, maintain a consistent volume of microcapsules, minimize sample loss, and preserve heat-sensitive components in FPOs [66].

Process parameters are crucial factors for the efficiency of freeze drying. For example, pressure can significantly impact both product quality and processing time. Under a freezing temperature of −10 °C, the use of high-pressure-induced freeze drying at 200 MPa resulted in more rounded and smooth microcapsules compared to those produced under pressures of 100 MPa and 300 MPa [67]. However, it should be noted that high pressures for a long period will lead to film rupture and oil phase outflow, so the appropriate conditions need to be based on the actual situation.

**Figure 4:**
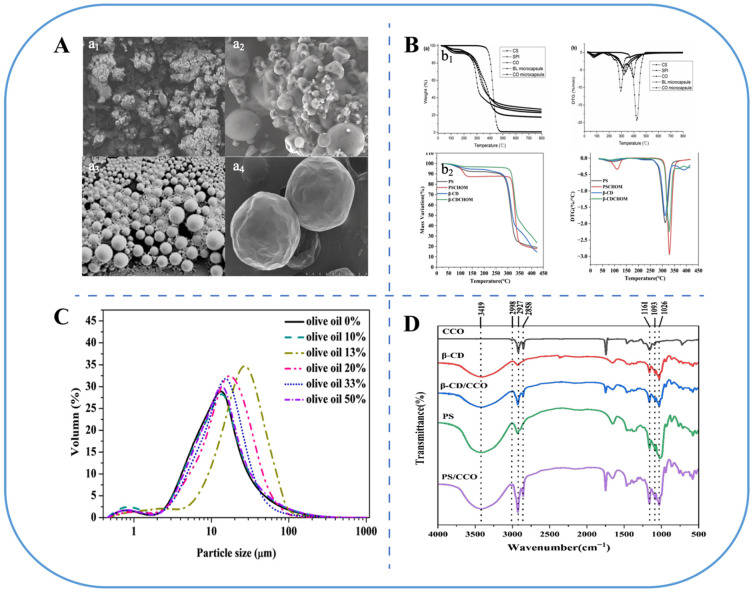
Characterization of microencapsulated FPOs. (**A**) scanning electron microscope (SEM) observes the morphology and structure of microcapsules: a_1_, freeze drying; a_2_, spray drying; a_3_, in situ polymerization; a_4_, complex coacervation [93,100,101,102]. (**B**) DSC (b_1_) and TGA (b_2_) analyses of thermal stability [69]. (**C**) Particle size analysis [103]. (**D**) FT-IR analysis [69].

Additionally, the freeze drying temperature serves as a crucial parameter in microcapsule preparation, as excessively low temperatures can result in oil droplet leakage. An increase in the particle size of microcapsules from 262.40 ± 4.16 nm to 353.73 ± 3.92 nm with freeze drying temperatures from −10 °C to −30 °C and a decrease in encapsulation efficiency from 87.6% to 60% compared to pre-freeze drying was observed [68]. The properties of freeze-dried microcapsules are typically characterized by differential scanning calorimetry (DSC)/thermogravimetric analysis (TGA) and Fourier Transform Infrared Spectroscopy (FT-IR). DSC and TGA demonstrated that coated Hickory oil (*Carya cathayensis Sarg*) with β-cyclodextrin (β-CD), porous starch (PS), and malt dextrin had excellent thermal stability at temperatures below 300 °C, indicating it was suitable for various food processing applications (Figure 4) [69].

At present, this technology can blend various wall materials such as alginate, gum arabic (GA), sodium caseinate (NaCas), lactose, maltodextrin (MD), and whey protein isolate/soybean protein isolate (SPI)) commonly used in the industry to prepare FPO (sunflower seed oil [70], flaxseed oil [71], chia seed oil [72], olive oil [73], etc.) microcapsules (Table 2). However, this approach is usually used in the processing of FPO microcapsules with high added value, owing to device complexity, time consumption, high energy consumption, and high maintenance costs [74].

#### 3.1.2. Spray Drying

Spray drying is a widely used technique for the preparation of microcapsules. Functional components are added to the appropriate wall material and homogenized to be encapsulated. The emulsion is fed into a spray dryer and atomized by a nozzle to vaporize the water and form a film on the surface of the droplets, which result in small particles being deposited on the bottom of the spray dryer; then, the microcapsules are transported to a cyclone separator for recycling (Figure 3) [75]. Generally, FPO microcapsules formed by spray drying had smoother surface morphology (Figure 4) and higher oxidation stability than freeze-dried microcapsules [76].

Optimization of inlet and outlet temperature is the key to preparing microcapsules by spray drying. When the inlet temperature of the spray dryer is too low, it will result in a reduction in water evaporation rate, leading to agglomerates or adhesion of microcapsules on the walls of the drying chamber. Conversely, excessive vaporization of the microcapsule may occur, causing production of cracks on its surface and premature release or degradation/loss of the encapsulated core material [61]. Conclusively, the cost of microcapsule products prepared by spray drying is lower than that of freeze drying, and there is a wide variety of products for microcapsules because of this technology having minimal thermal damage to sensitive materials, low loss rates, and excellent encapsulation effects [61,77,78].

### 3.2. Chemical Methods

Microcapsules could form an oil-in-water system through interfacial polymerization [79]. The water phase contains water-soluble monomers/initiators, while the oil phase consists of organic solvents, oil-soluble monomers, and core materials. The two phases react at the interface to ultimately form the microcapsule [80] (Figure 3). Notably, this method exhibits remarkable stability toward environmental variations. For instance, a highly polymerized polyurea shell layer was formed by interfacial imine chemistry of 1,6-hexane diamine—among them, olive oil is the core material and methylene diisocyanate is the wall material—in which adsorption capacity can reach 68 ± 1.0 mg/g [81]. However, the method of interfacial polymerization to prepare microcapsules is prone to some disadvantages, such as monomer residue, environmental pollution, and high cost, which limits its large-scale application in the food industry [82,83].

### 3.3. Physical–Chemical Methods

#### 3.3.1. Complex Coacervation

Complex coacervation is a unique and promising technique for FPO microencapsulation. The formation of complex coacervates is influenced by the type of polyelectrolyte, molecular weight, polyelectrolytes molar ratio, charge density, ionic strength, and processing conditions. At present, it can be divided into simple/complex condensation methods. Simple coacervation refers to the method of using two kinds of polymer materials with opposite charges as composite capsules, by stirring, centrifuging, and other methods to condense the core material into a shell. Complex composite condensation is more commonly used by adjusting the pH, temperature, and other conditions of the polymer material so that charges attract each other and form a complex, and then freeze drying/spray drying is used to wrap the material that needs to be protected and transferred, which can effectively protect the hydrophobic material from the external water phase and improve the interface activity (Figure 3) [65,84]. This method can not only better control the particle size (the diameter reach to the nanometer scale), drug loading, wall permeability, and release performance of microcapsules but also effectively improve the antioxidant activity of the product [85,86]. As a case, Wang et al. used sodium alginate (SA) and chitosan ammonium salt to obtain antibacterial microcapsules of citronella oil [87]. A combination of pectin and whey protein concentrate (WPC) created vitamin encapsulation with an encapsulation efficiency of 80.71% and a particle size reduction of 7.07 μm [88]. Peppermint oil microcapsules were prepared by using fungal chitosan, GA, and MD after complex coacervation and spray drying, and the storage period was extended to 50 days [89]. The synthesis of microcapsules via complex coacervation has gained increasing attention due to its low cost, ease of operation, high encapsulation efficiency, and stability [90]. However, this method exhibits a high sensitivity to variations in pH and ionic strength, posing challenges in maintaining the stability of core materials under extreme conditions [9].

#### 3.3.2. Molecular Embedding

Molecular embedding involves the incorporation of active ingredients by intermolecular crosslinking and utilizing specific protein groups and modifiers, which offers mild operating conditions and a straightforward procedure [91]. However, the use of materials including enzymes and cyclodextrins increases the economic cost, which needs to be considered. To facilitate the widespread implementation of immobilization technology in the food and pharmaceutical fields, continuous advancements are being made in the technique of molecular embedding. Ultrasonic-assisted molecular embedding (UME) technology harnesses the effects of cavitation, mechanical force, heat, and chemical reactions induced by ultrasonic waves, which significantly enhance encapsulation efficiency, providing promising prospects for practical applications [92]. UME technology encapsulate walnut oil with β-CD and PS as wall materials, achieving embedding rates of 80.40% and 75.52%, respectively [69] (Table 2). Hemp seed oil embedded in SPI and MD via UME technology can effectively reduce particle size, improve stability, and enhance encapsulation efficiency, accompanied with minimal flavor loss and maximum retention of heat-sensitive substances [80]. The UME technology, as a whole, not only enhances the production efficiency and quality of microcapsules but also effectively preserves the core material’s flavor [92].

#### 3.3.3. In Situ Polymerization

The microcapsules are formed through the in situ polymerization of emulsifiers and other additives on the surface of the core material, resulting in the formation of a membrane (Figure 3). This method possesses several advantages, including a straight forward process, cost-effectiveness, high density, and prolonged retention time for the microcapsules [93]. However, the application of this method is limited due to the environmental pollution caused by the microplastic polymer shell. In recent years, researchers have expanded the application of microcapsules in the food industry by developing natural extracts or changing the degree of crosslinking of phase change materials and controlling the directional arrangement on the surface of the core material droplet and the molecular weight of the polymer to make food additives, effectively preventing the active substance from direct contact with the outside world and protecting its activity [94]. Particularly, solid particles are irreversibly immobilized at the oil–water interface in Pickering emulsions, resulting in them being recognized as ideal templates for fabricating essential oil-loaded microcapsules [95,96,97,98]. Some researchers have used this method to prepare a cinnamon oil microcapsule, whose minimum inhibition concentration (MIC) against *E. coli* and *S. aureus* was 0.5 mg/mL, indicating a good antibacterial effect [99]. Compared to interfacial polymerization, this method is more economical due to reactants existing in the same phase [99].

**Table 2:** Microcapsule preparation techniques of different FPOs.

FPOs Name	Microencapsulation Process	Advantage	Wall Material	Encapsulation Efficiency	Reference
flaxseed oil	freeze drying	significantly improve antioxidant capacity and the quantities of PUFAs in products	SA-MD-inulin, soy protein concentrate	97.64%	[104]
	freeze drying	delay oil oxidation and prevent the production of primary oxidation products such as hydroperoxide free radicals	SPI	55.1%	[105]
	spray drying	retain volatile substances, the surface of powder particles has very low amount of unencapsulated oil, which improves excellent emulsification stability.	WPC, MD	86.98%	[106]
	spray drying	low price, it achieves high microencapsulation efficiency and good oxidation stability under high oil load.	NaCas, isomaltooligosaccharide	98.22%	[107]
chia seed oil	freeze drying	minimize the damage caused by temperature to the product, the oxidation stability of chia oil was significantly improved	NaCas, lactose	85%	[72]
	spray drying	effectively protects chia seed oil from oxidation processes.	bean protein isolate and peach gum	96%	[108]
	spray drying	effectively prevent lipid oxidation.	sunflower lecithins, chitosan, chia mucilage, MD	99.11%	[109]
	spray drying	prevent lipid oxidation.	SPI, MD, inulin	88.79%	[110]
	complex coacervation	high load, high encapsulation efficiency, mild processing conditions.	CPI-CGS	93.9%	[111]
soybean oil	spray drying	a more effective interfacial barrier is generated, which improves the thermal stability and oxidation stability of the microencapsulated oil	MD	93.41%	[112]
	situ polymerization	provide good adhesion.	urea-formaldehyde	75%	[113]
corn oil	spray drying	protect bioactive compounds that are susceptible to degradation along the human digestive tract, low price.	brea gum, GA, inulin	91.72%	[114]
sesame oil	complex coacervation	the oxidative stability was remarkably enhanced	gelatin, GA	90.25%	[41]
sunflower seed oil	spray drying	improve oxidative stability.	pea protein isolate	88%	[115]
sea buckthorn oil	spray drying	extend the shelf life.	SPI, soybean polysaccharide	95.30%	[116]
almond oil	spray drying	protection against oxidation reactions.	taro starch	56%	[117]
perilla oil	complex coacervation	better polydispersity index, encapsulation efficiency, and oxidative stability.	OSA-starch	98.18%	[118]
peppermint oil	complex coacervation	effectively extend the storage period	fungal chitosan, GA, MD	29%	[89]
	spray drying	limit loss and degradation of flavors and aromas during processing and storage	bovine serum albumin and GA	54%	[119]
	freeze drying	extend the oxidative	GA, MD	91%	[120]
vanilla oil	spray drying	improve product flavor and antioxidant properties	Chitosan, GA	69.20%	[121]
olive oil	spray drying	increase oil stability and reduce digestibility.	octenyl succinic anhydride rice starch	93.14%	[122]
	spray drying	limit the oxidation of grease.	sour cherry protein isolate	97.71%	[123]
	complex coacervation	improve thermal stability.	gelatin, SA	89.37%	[124]
coconut oil	interfacial polymerization	improve thermal stability.	SA	81.1%	[125]
	spray drying	improve antioxidant activity.	inulin	88.10%	[126]
walnut oil	spray drying	improve thermal stability.	SPI, MD	72.2%	[127]
	freeze drying	the water content and hygroscopicity of microcapsules were reduced.	fructooligosaccharide, SPI	60%	[128]
	UME	Improve the oxidation stability of vegetable oil	β-CD and PS	80.40%, 75.52%	[69]
hazelnut oil	spray drying	prolong the shelf life of the nuts.	hydroxypropyl methylcellulose, MD	81.6%	[129]
pine-seed oil	complex coacervation, freeze drying	better resistance to high temperature, high humidity and controlled release characteristics.	gelatin, GA, MD	80.87%	[130]
	spray drying	Enhance the thermal stability and antioxidant activity of the oil, microcapsule can be added to the food substrate as a food preservative	GA, MD	70.07%	[131]
zanthoxylum oil	complex coacervation	improve the thermal stability of microcapsules.	chitosan-gelatin	60.05%	[132]
	UME	improve encapsulation efficiency, make the microcapsules smaller and more evenly in size, it can also preserve the flavor of the core for alonger period of time and even give the core some new chemical properties.	β-CD	81.94%	[6]
	complex coacervation, spray drying	low price, simplicity of operator, high encapsulation efficiency, high temperature resistance and humidity stability.	quinoa protein isolate-GA coacervates, sodium tripolyphosphate	87.25%	[90]
TTO	complex coacervation	simple operation and environmental protection, effectively improve the stability of TTO and prolong its bacteriostatic effect.	alginate, quaternary ammonium salt of chitosan	66.06%	[85]
	spray drying	good emulsifying property, improved oil stability and concentration in the powder.	chitosan, alginate	90.4%	[77]
	spray drying	the oxidation stability and retention of PUFAs were improved.	mung bean protein isolate, alginate	72.09%	[133]
	interfacial polymerization	the volatile liquid component is retained to a large extent.	alginate calcium	97.5%	[134]
	in situ polymerization	the microcapsules are not agglomerate.	urea-formaldehyde	45%	[135]
	freeze drying	oxidation stability is significantly improved.	WPC, MD, SSOS	95.17%	[136]
	freeze drying	improved heat resistance, the peroxide value decreased significantly and oxidation stability increased.	chitosan, SPI	87%	[100]
	freeze drying	good water solubility, Oxidation stability is significantly improved.	MD	66.41%	[137]
peony seed oil	spray drying	extend the shelf life, enhanced thermal stability.	SSOS, β-CD, Pectin	92.5%	[138]
	spray drying	high encapsulation efficiency and oxidation stability, less loss of active substance.	whey protein, corn syrup (CS), soya bean lecithin	80%	[139]
	spray drying	low price, high encapsulation efficiency, it has excellent film forming ability and emulsifying property.	NaCas, CS	93.71%	[140]
	spray drying	high encapsulation efficiency, effectively prevent lipid oxidation, the wall material has high solubility, low viscosity, good emulsifying property.	GA, CS, NaCas	92.8%	[141]

UME: ultrasonic-assisted molecular embedding, PUFA: polyunsaturated fatty acid, TTO: tea tree oil, SA: sodium alginate, MD: maltodextrin, SPI: soybean protein isolate, NaCas: sodium caseinate, CPI-CGS: chia seed protein isolate- chia seed gum, GA: gum Arabic, WPC: whey protein concentrate, SSOS: sodium starch octenyl succinate, β-CD: β-cyclodextrin, CS: corn syrup, OSA-starch: octenyl succinic anhydride starch.

## 4. Applications of FPO Microcapsules in the Food Industry

**Figure 5:**
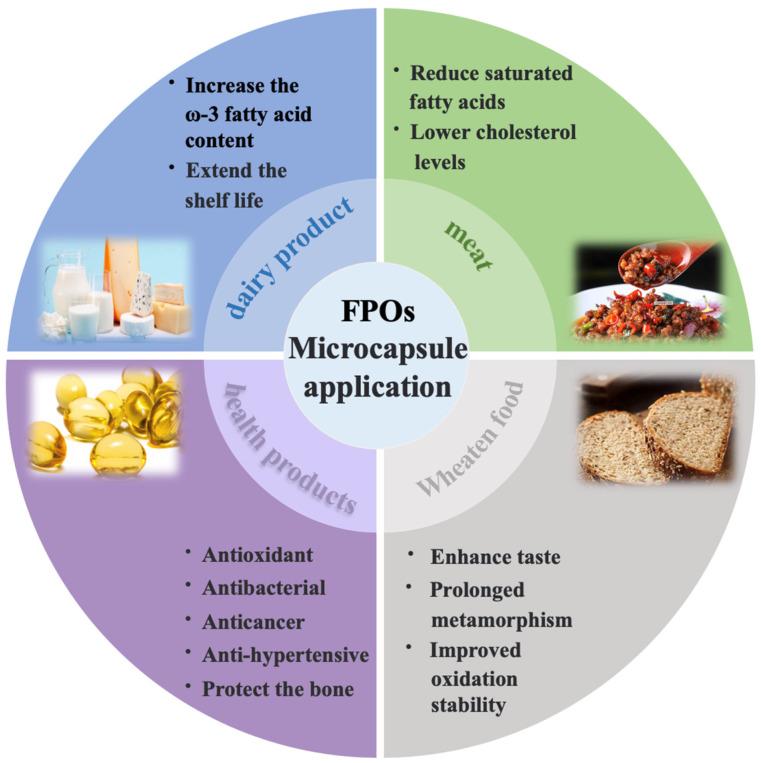
Application of microcapsules in functional foods.

FPOs have gained significant attention due to their ability to efficiently provide energy and essential fatty acids, as well as enhance the absorption of fat-soluble nutrients [142]. Therefore, an increasing number of researchers continuously explore applications of FPOs in the food industry based on it has antibacterial and antioxidation effects, and reduction in additive usage, etc. Currently, microencapsulation techniques have exhibited remarkable efficacy in incorporating FPOs into various food products. This technique can alter the physical properties of foods, delay lipid oxidation, improve overall nutrient retention, serve as natural preservatives, and enhance flavor profiles (Figure 5) [143,144,145,146].

### 4.1. Changing the Physical Properties of Foods

Microencapsulation is feasible to transform FPOs from a liquid state into minute solid particles, which retards the reaction between oil and oxygen to enhance oxidation stability [147]. Hydrophilic groups in the shell material, such as carboxyl, hydroxyl, and amino groups, form a solution or gel by combining with water, which plays a role in stabilizing emulsification and enhancing adhesion and elasticity in food processing. Microcapsules composed of hydrophobic groups composed of fatty acid chains can effectively prevent food from absorbing water, maintain the hardness and elasticity of food, and effectively extend the shelf life of fatty food. The suitable shell material is selected according to the nature of the embedded core material during use to maintain the hydrophilic lipophilic balance (HLB) of food materials [148]. For example, meats are susceptible to oxidation, leading to spoilage. The addition of FPO microcapsules effectively addresses this issue while improving the color and quality of meat products. A 9-point hedonic scale demonstrated that the color and odor ratings for microcapsules were consistently above 6.3 over a period of 15 days [149]. The addition of flaxseed oil microcapsules prepared by spray drying sausage can enhance its water-holding capacity by 10.3% compared to direct oil addition and increase the sausage’s hardness and elasticity [150]. Studies have demonstrated that incorporating 5.2 g of chia seed oil microcapsules into a dough weighing (100 g) can provide a significant content of omega-3 fatty acids, decreasing volume and increasing hardness, color saturation, aroma intensity, and overall acceptance of bread [151]. Furthermore, some researchers have utilized microencapsulation to mix walnut oil or pistachio oil to the paste (15 g/100 g) as a substitute for animal fat, because this technique could alter paste’s physical and chemical properties [152].

### 4.2. Delaying the Oxidation of Lipids

The stable wall material effectively prevents the FPOs in the microcapsule from contacting the outside environment, thereby delaying lipid oxidation and extending food shelf life. When flaxseed oil and probiotics were coated with different wall materials and then added to yogurt, the PV of yogurt within 28 days (3.13–3.83 meq O_2_/kg) was significantly lower than that of uncoated flaxseed oil (8.62 meq O_2_/kg) [104]. The addition of chia seed oil microcapsules to dry pasta by manufacturers serves as a preventive measure against spoilage and hampered the generation of hydrogen peroxide free radicals throughout the stages of preparation, storage, and cooking, thereby enhancing product safety [105]. The researchers discovered that its addition to bread had no impact on its taste while effectively inhibiting the formation of hydroperoxide free radicals, thereby enhancing product safety [145]. Microcapsules containing garlic oil or a combination of flaxseed oil and graphene oxide can be incorporated into bread to promote human health and ensure the presence of high-quality omega-3 fatty acids [145]. The utilization of sodium starch octenyl succinate (SSOS), β-CD, and pectin as wall materials in the microencapsulation of peony seed oil, a novel food additive, has been proven to effectively extend its shelf life [138,153]. Consequently, microencapsulation techniques can significantly enhance the chemical stability of FPOs, thereby offering valuable insights for their diverse applications within the food industry.

### 4.3. Improving Overall Nutrient Retention

FPO microcapsules can be incorporated into various food products to enhance their unsaturated fatty acid content, thereby improving their nutritional comprehensiveness and functionality. For example, the infusion of flaxseed oil into chicken sausages enables an increase in ALA [150]. The incorporation of microcapsules containing flaxseed oil into milk represents an efficient strategy for augmenting the omega-3 fatty acid content in both milk and pasta [105]. Comparable findings were observed in a ready-to-eat salad formulated with microcapsules encapsulating olive oil [73]. The oxidative stability of Graham biscuits prepared using TTO microcapsules was significantly higher, resulting in a greater content of PUFAs [133]. Within a period of 6 weeks, the peroxide value (PV) of the microcapsules was observed to be lower than that of the free oil. Incorporating microcapsules into biscuits at a concentration of 10% resulted in improved crispness and enhanced shine, indicating that the embedding tool enhances physiological attributes such as color and storage [133]. Therefore, microcapsules made of FPOs not only serve as a barrier against oil oxidation and can protect volatile substances, but more importantly, effectively retain a higher content of fatty acids, thereby enhancing the nutritional value of food.

### 4.4. Natural Preservatives

The antibacterial and preservative effects of microcapsules containing FPOs are also being gradually explored in the food industry. Pepper essential oil was encapsulated through the spray drying of double-layer emulsions (SPI/high methoxyl pectin), which exhibited inhibitory effects on the growth of *S. aureus* and *L. monocytogenes* of milk [133]. Furthermore, the incorporation of FPO microcapsules into milk can inhibit the growth of *Staphylococcus aureus*, *Bacillus subtilis*, *Listeria monocytogenes*, and *Listeria innocua*, highlighting their potential as a natural preservative in the food industry [133]. Similarly, the addition of zanthoxylum oil microcapsules can effectively extend the induction period of lipid oxidation and prevent microbial proliferation in the short term, indicating its promising application as a natural additive for maintaining the quality of rabbit meat patties [154]. In general, microcapsules derived from FPOs can serve as a favorable choice of natural materials for food preservation.

### 4.5. Enriching the Flavor of Foods

Microcapsules containing flavored FPOs can be utilized to enhance food flavor or reduce the release of unpleasant odors. For instance, argan oil microcapsule powder produced through spray-coating with GA, MD, and gelatin can be incorporated into daily meals to augment the aromatic profile of lipid-based foods [155]. The high content of PUFAs in nut oils produced beneficial effects on human health. After microencapsulation of nut oils, the water activity remained below 0.24, and the peroxidation index of was consistently low (less than 15 meq/kg), which inhibited the production of odor and enhanced nutrient retention [152]. In summary, microencapsulated FPOs not only preserved the original food quality but also mitigated undesirable flavors, which overcame the drawback of oxidation-induced taste deterioration.

## 5. Conclusions and Future Trends for FPOs Microcapsules

In this study, we have systematically summarized the classification, nutritional composition, microcapsule preparation tools, and application fields of FPO microcapsules. FPO microcapsules have gained widespread approval and application as nutritional and functional materials in the food industry. However, there are emerging development trends and challenges in the following aspects:

(a) Increased availability of wall materials: The selection of wall materials plays a crucial role in encapsulation efficiency of FPO microcapsules. Researchers should actively seek low-cost, excellent emulsification properties, and superior oil encapsulation capabilities materials. Notably, natural wall materials with enhanced embedding effects and greater health benefits are an important area of research focus;
(b) Advanced techniques for microcapsule preparation: The integration of multiple preparation techniques, such as complex coacervation in combination with interfacial polymerization and freeze drying coupled with spray drying, has the potential to enhance the performance of microcapsule products;
(c) Broader spectrum of microcapsule dimensions: Currently, the size distribution of FPO microcapsules typically ranges from 1 to 1000 μm, following a normal distribution (Figure 3) [103]. In fact, as advancements are made in emulsification techniques, FPO microcapsules will exhibit more uniformity in size and regular shapes;
(d) Application for supplementary fields: Microcapsules could offer a novel avenue for FPO development, such as enhancing the flavor and quality of prepared dishes or vegetarian meats. Additionally, microencapsulated FPOs exhibit resistance to oxidation and serve as an important natural source for cosmeceuticals.

## Data Availability

The original contributions presented in the study are included in the article, further inquiries can be directed to the corresponding author.

## References

[1] Kotecka-Majchrzak K., Sumara A., Fornal E., Montowska M. (2020). Oilseed Proteins—Properties and Application as a Food Ingredient. Trends Food Sci. Technol..

[2] Gharibzahedi S.M.T., Smith B. (2021). Legume Proteins Are Smart Carriers to Encapsulate Hydrophilic and Hydrophobic Bioactive Compounds and Probiotic Bacteria: A Review. Comp. Rev. Food Sci. Food Saf..

[3] Janik M., Hanula M., Khachatryan K., Khachatryan G. (2023). Nano-/Microcapsules, Liposomes, and Micelles in Polysaccharide Carriers: Applications in Food Technology. Appl. Sci..

[4] Zhu H., Zhang Y., Tian J., Chu Z. (2018). Effect of a New Shell Material—Jackfruit Seed Starch on Novel Flavor Microcapsules Containing Vanilla Oil. Ind. Crops Prod..

[5] He R., Ye J., Wang L., Sun P. (2020). Preparation and Evaluation of Microcapsules Encapsulating Royal Jelly Sieve Residue: Flavor and Release Profile. Appl. Sci..

[6] Li J., Hou X., Jiang L., Xia D., Chen A., Li S., Li Q., Gu X., Mo X., Zhang Z. (2022). Optimization and Characterization of Sichuan Pepper (*Zanthoxylum bungeanum* Maxim) Resin Microcapsule Encapsulated with β-Cyclodextrin. LWT.

[7] Zhang X., Wang D., Liu L., Jiang W., Xiang W., Zhang Q., Tang J. (2024). Microencapsulation of Zanthoxylum Schinifolium Essential Oil through Emulsification Followed by Spray Drying: Microcapsule Characterization and Functional Evaluation. Colloids Surf. A Physicochem. Eng. Asp..

[8] Pattnaik M., Mishra H.N. (2022). Amelioration of the Stability of Polyunsaturated Fatty Acids and Bioactive Enriched Vegetable Oil: Blending, Encapsulation, and Its Application. Crit. Rev. Food Sci. Nutr..

[9] Timilsena Y.P., Akanbi T.O., Khalid N., Adhikari B., Barrow C.J. (2019). Complex Coacervation: Principles, Mechanisms and Applications in Microencapsulation. Int. J. Biol. Macromol..

[10] Tan C.X. (2019). Virgin Avocado Oil: An Emerging Source of Functional Fruit Oil. J. Funct. Foods.

[11] Deng L., Yuan H., Xie J., Ge L., Chen Y. (2022). Herbaceous Plants Are Better than Woody Plants for Carbon Sequestration. Resour. Conserv. Recycl..

[12] Čeh B., Štraus S., Hladnik A., Kušar A. (2020). Impact of Linseed Variety, Location and Production Year on Seed Yield, Oil Content and Its Composition. Agronomy.

[13] Kim H.U., Lee K.-R., Jeon I., Jung H.E., Heo J.B., Kim T.-Y., Chen G.Q. (2019). Fatty Acid Composition and Oil Content of Seeds from Perilla (*Perilla frutescens* (L.) var. *frutescens*) Germplasm of Republic of Korea. Genet. Resour. Crop Evol..

[14] Timilsena Y.P., Vongsvivut J., Adhikari R., Adhikari B. (2017). Physicochemical and Thermal Characteristics of Australian Chia Seed Oil. Food Chem..

[15] Romanić R.S., Lužaić T.Z., Radić B.Đ. (2021). Enriched Sunflower Oil with Omega 3 Fatty Acids from Flaxseed Oil: Prediction of the Nutritive Characteristics. LWT.

[16] Abdullah S., Asif M., Ali H., Ali R., Saleem M. (2022). Characterization of Corn Oil Using Fluorescence Spectroscopy. J. Fluoresc..

[17] Gabay O., Sanchez C., Salvat C., Chevy F., Breton M., Nourissat G., Wolf C., Jacques C., Berenbaum F. (2010). Stigmasterol: A Phytosterol with Potential Anti-Osteoarthritic Properties. Osteoarthr. Cartil..

[18] Aksoz E., Korkut O., Aksit D., Gokbulut C. (2020). Vitamin E (A-, β + Γ- and Δ-tocopherol) Levels in Plant Oils. Flavour Fragr. J..

[19] Wu H., Xu L., Ballantyne C.M. (2020). Dietary and Pharmacological Fatty Acids and Cardiovascular Health. J. Clin. Endocrinol. Metab..

[20] Psota T.L., Gebauer S.K., Kris-Etherton P. (2006). Dietary Omega-3 Fatty Acid Intake and Cardiovascular Risk. Am. J. Cardiol..

[21] Alessandri C., Pignatelli P., Loffredo L., Lenti L., Del Ben M., Carnevale R., Perrone A., Ferro D., Angelico F., Violi F. (2006). Alpha-Linolenic Acid–Rich Wheat Germ Oil Decreases Oxidative Stress and CD40 Ligand in Patients with Mild Hypercholesterolemia. Arterioscler. Thromb. Vasc. Biol..

[22] Zhang Y., Dong L., Yang X., Shi H., Zhang L. (2011). α-Linolenic Acid Prevents Endoplasmic Reticulum Stress-Mediated Apoptosis of Stearic Acid Lipotoxicity on Primary Rat Hepatocytes. Lipids Health Dis..

[23] Upadhyay R., Mishra H.N. (2015). Predictive Modeling for Shelf Life Estimation of Sunflower Oil Blended with Oleoresin Rosemary (*Rosmarinus officinalis* L.) and Ascorbyl Palmitate at Low and High Temperatures. LWT-Food Sci. Technol..

[24] Alencar S.A.D.S., Kiefer C., Nascimento K.M.R.D.S., Viana L.H., Corassa A., Gomes M.D.N.B., Marçal D.A., Farias T.V.A. (2021). Dietary Soybean Oil Modulates Fatty Acid Composition of Pork. Trop. Anim. Health Prod..

[25] Szewczyk K., Górnicka M. (2023). Dietary Vitamin E Isoforms Intake: Development of a New Tool to Assess Tocopherols and Tocotrienols Intake in Adults. Nutrients.

[26] Zhao Y., Wang Y., Zhang Z., Li H. (2023). Advances in Controllable Release Essential Oil Microcapsules and Their Promising Applications. Molecules.

[27] Flakelar C.L., Adjonu R., Doran G., Howitt J.A., Luckett D.J., Prenzler P.D. (2022). Phytosterol, Tocopherol and Carotenoid Retention during Commercial Processing of *Brassica napus* (Canola) Oil. Processes.

[28] Yu J., Yan H., Wu Y., Wang Y., Xia P. (2022). Quality Evaluation of the Oil of *Camellia* spp. Foods.

[29] Drehmer E., Navarro-Moreno M.Á., Carrera-Juliá S., Moreno M.L. (2022). A Comparative Study between Olive Oil and Corn Oil on Oxidative Metabolism. Food Funct..

[30] Lee C.-P., Shih P.-H., Hsu C.-L., Yen G.-C. (2007). Hepatoprotection of Tea Seed Oil (*Camellia oleifera* Abel.) against CCl_4_-Induced Oxidative Damage in Rats. Food Chem. Toxicol..

[31] de Paiva Azevedo E.P., dos Santos Alves E.M., de Souza J.R.B., de Araújo K.S., de Santana Khan S., de Mendonça C.E.A., Maciel M.I.S. (2021). Fatty Acid in Raw and Heated Coconut Oil in Eleven Coconut Oil Food Preparations Analysed by Gas Chromatography. Int. J. Gastron. Food Sci..

[32] Hernández M.L., Muñoz-Ocaña C., Posada P., Sicardo M.D., Hornero-Méndez D., Gómez-Coca R.B., Belaj A., Moreda W., Martínez-Rivas J.M. (2023). Functional Characterization of Four Olive Squalene Synthases with Respect to the Squalene Content of the Virgin Olive Oil. J. Agric. Food Chem..

[33] Amarowicz R. (2009). Squalene: A Natural Antioxidant?. Eur. J. Lipid Sci. Technol..

[34] Zhang Y.-Y., Zhang F., Thakur K., Ci A.-T., Wang H., Zhang J.-G., Wei Z.-J. (2018). Effect of Natural Polyphenol on the Oxidative Stability of Pecan Oil. Food Chem. Toxicol..

[35] Al-Madhagy S., Ashmawy N.S., Mamdouh A., Eldahshan O.A., Farag M.A. (2023). A Comprehensive Review of the Health Benefits of Flaxseed Oil in Relation to Its Chemical Composition and Comparison with Other Omega-3-Rich Oils. Eur. J. Med. Res..

[36] Dąbrowski G., Konopka I., Czaplicki S. (2018). Variation in Oil Quality and Content of Low Molecular Lipophilic Compounds in Chia Seed Oils. Int. J. Food Prop..

[37] Gopalam R., Manasa V., Vaishnav S.R., Daga P., Tumaney A.W. (2022). Profiling of Lipids, Nutraceuticals, and Bioactive Compounds Extracted from an Oilseed Rich in PUFA. Plant Foods Hum. Nutr..

[38] Kim K.-B., Nam Y.A., Kim H.S., Hayes A.W., Lee B.-M. (2014). α-Linolenic Acid: Nutraceutical, Pharmacological and Toxicological Evaluation. Food Chem. Toxicol..

[39] Li Z., Sun B., Zhu Y., Liu L., Huang Y., Lu M., Zhu X., Gao Y. (2022). Effect of Maltodextrin on the Oxidative Stability of Ultrasonically Induced Soybean Oil Bodies Microcapsules. Front. Nutr..

[40] Jumina J., Lavendi W., Singgih T., Triono S., Steven Kurniawan Y., Koketsu M. (2019). Preparation of Monoacylglycerol Derivatives from Indonesian Edible Oil and Their Antimicrobial Assay against *Staphylococcus aureus* and *Escherichia coli*. Sci. Rep..

[41] Dai H.-H., Li X.-D., Wei A.-C., Wang X.-D., Wang D.-Y. (2020). Characterization and Oxidative Stability of Cold-Pressed Sesame Oil Microcapsules Prepared by Complex Coacervation. J. Oleo Sci..

[42] Parsaeian M., Shahabi M., Hassanpour H. (2020). The Integration of Image Processing and Artificial Neural Network to Estimate Four Fatty Acid Contents of Sesame Oil. LWT.

[43] Pérez-Vich B., Garcés R., Fernández-Martínez J.M. (2000). Epistatic Interaction among Loci Controlling the Palmitic and the Stearic Acid Levels in the Seed Oil of Sunflower. Theor. Appl. Genet..

[44] Petraru A., Ursachi F., Amariei S. (2021). Nutritional Characteristics Assessment of Sunflower Seeds, Oil and Cake. Perspective of Using Sunflower Oilcakes as a Functional Ingredient. Plants.

[45] Li Z., Wang J., Xiong Y., Li Z., Feng S. (2016). The Determination of the Fatty Acid Content of Sea Buckthorn Seed Oil Using near Infrared Spectroscopy and Variable Selection Methods for Multivariate Calibration. Vib. Spectrosc..

[46] Górnaś P., Rudzińska M. (2016). Seeds Recovered from Industry By-Products of Nine Fruit Species with a High Potential Utility as a Source of Unconventional Oil for Biodiesel and Cosmetic and Pharmaceutical Sectors. Ind. Crops Prod..

[47] Kesen S., Amanpour A., Selli S. (2018). Comparative Evaluation of the Fatty Acids and Aroma Compounds in Selected Iranian Nut Oils. Eur. J. Lipid Sci. Technol..

[48] Domingues P.M., Santos L. (2019). Essential Oil of Pennyroyal (*Mentha pulegium*): Composition and Applications as Alternatives to Pesticides—New Tendencies. Ind. Crops Prod..

[49] Sinha A.K., Sharma U.K., Sharma N. (2008). A Comprehensive Review on Vanilla Flavor: Extraction, Isolation and Quantification of Vanillin and Others Constituents. Int. J. Food Sci. Nutr..

[50] Wang Y., Li B., Zhu L., Wang P., Xu F., Zhang Y. (2022). Octenyl Succinic Acid Starch-Stabilized Vanilla Essential Oil Pickering Emulsion: Preparation, Characterization, Antioxidant Activity, and Storage Stability. Foods.

[51] Gunduz G., Konuskan D.B. (2023). Fatty Acid and Sterol Compositions of Turkish Monovarietal Olive Oils with Regard to Olive Ripening. J. Oleo Sci..

[52] Al-Bachir M., Sahloul H. (2017). Fatty Acid Profile of Olive Oil Extracted from Irradiated and Non-Irradiated Olive Fruits. Int. J. Food Prop..

[53] Devi A., Khatkar B.S. (2018). Effects of Fatty Acids Composition and Microstructure Properties of Fats and Oils on Textural Properties of Dough and Cookie Quality. J. Food Sci. Technol..

[54] Eyres L., Eyres M.F., Chisholm A., Brown R.C. (2016). Coconut Oil Consumption and Cardiovascular Risk Factors in Humans. Nutr. Rev..

[55] Adhikari P., Zhu X.-M., Gautam A., Shin J.-A., Hu J.-N., Lee J.-H., Akoh C.C., Lee K.-T. (2010). Scaled-up Production of Zero-Trans Margarine Fat Using Pine Nut Oil and Palm Stearin. Food Chem..

[56] Matthäus B., Li P., Ma F., Zhou H., Jiang J., Özcan M.M. (2018). Is the Profile of Fatty Acids, Tocopherols, and Amino Acids Suitable to Differentiate *Pinus armandii* Suspicious to Be Responsible for the Pine Nut Syndrome from Other *Pinus* Species?. Chem. Biodivers..

[57] Xu Y.X., Hanna M.A. (2010). Evaluation of Nebraska Hybrid Hazelnuts: Nut/Kernel Characteristics, Kernel Proximate Composition, and Oil and Protein Properties. Ind. Crops Prod..

[58] Zhang B., Luo S., Wu S., Li J., He F., Luo R., Wu Y., Pan M., Pei X., Yong L. (2023). Anti-Inflammatory and Anti-Osteoclastogenesis Activities of Different Kinds of *Zanthoxylum bungeanum* Seed Oil in Vitro. Chem. Biodivers..

[59] Fei X., Ma Y., Hu H., Wei A. (2020). Transcriptome Analysis and GC-MS Profiling of Key Genes in Fatty Acid Synthesis of *Zanthoxylum bungeanum* Seeds. Ind. Crops Prod..

[60] Shi T., Wu G., Jin Q., Wang X. (2020). Camellia Oil Authentication: A Comparative Analysis and Recent Analytical Techniques Developed for Its Assessment. A Review. Trends Food Sci. Technol..

[61] Kurt O., Göre M. (2020). Effects Of Sowing Date and Genotype on Oil Content and Main Fatty Acid Composition in Camelina [*Camelina sativa* L. (Crantz)]. Turk. J. Field Crops.

[62] Qiu S., Wang X., Zan M., Wang Z., Dang L. (2021). The Insight into Separation of Oleic, Linoleic, and α-Linolenic Acid in Peony Seed Oil from Eutectic Behaviors, Polymorphic Transition and Solid-Liquid Phase Equilibrium. LWT.

[63] Liu H., Wang L., Yang T., Zhang G., Huang J., Sun J., Huo J. (2016). Optimization and Evaluation of Fish Oil Microcapsules. Particuology.

[64] Ozkan G., Franco P., De Marco I., Xiao J., Capanoglu E. (2019). A Review of Microencapsulation Methods for Food Antioxidants: Principles, Advantages, Drawbacks and Applications. Food Chem..

[65] Muhoza B., Yuyang H., Uriho A., Harindintwali J.D., Liu Q., Li Y. (2023). Spray-and Freeze-Drying of Microcapsules Prepared by Complex Coacervation Method: A Review. Food Hydrocoll..

[66] Yakdhane A., Labidi S., Chaabane D., Tolnay A., Nath A., Koris A., Vatai G. (2021). Microencapsulation of Flaxseed Oil—State of Art. Processes.

[67] Choi M.-J., Hong G.-P., Briançon S., Fessi H., Lee M.-Y., Min S.-G. (2008). Effect of a High-Pressure-Induced Freezing Process on the Stability of Freeze-Dried Nanocapsules. Dry. Technol..

[68] Bejrapha P., Min S.-G., Surassmo S., Choi M.-J. (2010). Physicothermal Properties of Freeze-Dried Fish Oil Nanocapsules Frozen under Different Conditions. Dry. Technol..

[69] Li Q., Wang L., Zheng M., Lu H., Liu Y., Wang Y., Lu S. (2023). Microencapsulation with Different Starch-Based Polymers for Improving Oxidative Stability of Cold-Pressed Hickory (*Carya cathayensis* Sarg). Oil. Foods.

[70] Gökkaya Erdem B., Kaya S. (2022). Edible Film Fabrication Modified by Freeze Drying from Whey Protein Isolate and Sunflower Oil: Functional Property Evaluation. Food Packag. Shelf Life.

[71] Elik A., Koçak Yanık D., Göğüş F. (2021). A Comparative Study of Encapsulation of Carotenoid Enriched-Flaxseed Oil and Flaxseed Oil by Spray Freeze-Drying and Spray Drying Techniques. LWT.

[72] Rodriguez E.S., Julio L.M., Henning C., Diehl B.W., Tomás M.C., Ixtaina V.Y. (2019). Effect of Natural Antioxidants on the Physicochemical Properties and Stability of Freeze-Dried Microencapsulated Chia Seed Oil: Freeze-Dried Microparticles with Chia Seed Oil and Natural Antioxidants. J. Sci. Food Agric..

[73] Silva K.A., Coelho M.A.Z., Calado V.M.A., Rocha-Leão M.H.M. (2013). Olive Oil and Lemon Salad Dressing Microencapsulated by Freeze-Drying. LWT-Food Sci. Technol..

[74] Díaz-Maroto M., Pérez-Coello M., Cabezudo M. (2002). Effect of Different Drying Methods on the Volatile Components of Parsley (*Petroselinum crispum* L.). Eur. Food Res. Technol..

[75] Maroof K., Lee R.F.S., Siow L.F., Gan S.H. (2022). Microencapsulation of Propolis by Spray Drying: A Review. Dry. Technol..

[76] Kaushik P., Dowling K., McKnight S., Barrow C.J., Adhikari B. (2016). Microencapsulation of Flaxseed Oil in Flaxseed Protein and Flaxseed Gum Complex Coacervates. Food Res. Int..

[77] Zhang S., Chen J., Yin X., Wang X., Qiu B., Zhu L., Lin Q. (2017). Microencapsulation of Tea Tree Oil by Spray-Drying with Methyl Cellulose as the Emulsifier and Wall Material Together with Chitosan/Alginate: ARTICLE. J. Appl. Polym. Sci..

[78] Bartella L., Di Donna L., Napoli A., Sindona G., Mazzotti F. (2019). High-Throughput Determination of Vitamin E in Extra Virgin Olive Oil by Paper Spray Tandem Mass Spectrometry. Anal. Bioanal. Chem..

[79] Yashin V.V., Balazs A.C. (2004). Theoretical Model of Interfacial Polymerization. J. Chem. Phys..

[80] Xiao Y., Wu B., Fu X., Wang R., Lei J. (2019). Preparation of Biodegradable Microcapsules through an Organic Solvent-Free Interfacial Polymerization Method. Polym. Adv. Technol..

[81] Wei Z., Ma X., Wang P., Pan J. (2022). Interfacial Imide Polymerization of Functionalized Filled Microcapsule Templates by the Pickering Emulsion Method for the Rapid Removal of 3,4,5-Trichlorophenol from Wastewater. Nanomaterials.

[82] Liao Z., Xue D., Li H., Shi L. (2016). Fragrance-Containing Microcapsules Based on Interfacial Thiol-Ene Polymerization. J. Appl. Polym. Sci..

[83] Podshivalov A.V., Bronnikov S., Zuev V.V., Jiamrungraksa T., Charuchinda S. (2013). Synthesis and Characterization of Polyurethane–Urea Microcapsules Containing Galangal Essential Oil: Statistical Analysis of Encapsulation. J. Microencapsul..

[84] Glomm W.R., Molesworth P.P., Yesiltas B., Jacobsen C., Johnsen H. (2023). Encapsulation of Salmon Oil Using Complex Coacervation: Probing the Effect of Gum Acacia on Interfacial Tension, Coacervation and Oxidative Stability. Food Hydrocoll..

[85] Chen M., Hu Y., Zhou J., Xie Y., Wu H., Yuan T., Yang Z. (2016). Facile Fabrication of Tea Tree Oil-Loaded Antibacterial Microcapsules by Complex Coacervation of Sodium Alginate/Quaternary Ammonium Salt of Chitosan. RSC Adv..

[86] Mei L., Ji Q., Jin Z., Guo T., Yu K., Ding W., Liu C., Wu Y., Zhang N. (2022). Nano-Microencapsulation of Tea Seed Oil via Modified Complex Coacervation with Propolis and Phosphatidylcholine for Improving Antioxidant Activity. LWT.

[87] Wang J., Li X., Chen M., Chen Z., Wu H., Zhang P., Yuan T., Yang Z., Hu Y. (2018). Fabrication of Sustained-Release and Antibacterial Citronella Oil-Loaded Composite Microcapsules Based on Pickering Emulsion Templates. J. Appl. Polym. Sci..

[88] Akbari N., Assadpour E., Kharazmi M.S., Jafari S.M. (2022). Encapsulation of Vitamin B12 by Complex Coacervation of Whey Protein Concentrate–Pectin; Optimization and Characterization. Molecules.

[89] Baiocco D., Preece J.A., Zhang Z. (2021). Microcapsules with a Fungal Chitosan-Gum Arabic-Maltodextrin Shell to Encapsulate Health-Beneficial Peppermint Oil. Food Hydrocoll. Health.

[90] Chen K., Zhang M., Mujumdar A.S., Wang M. (2023). Quinoa Protein Isolate-Gum Arabic Coacervates Cross-Linked with Sodium Tripolyphosphate: Characterization, Environmental Stability, and Sichuan Pepper Essential Oil Microencapsulation. Food Chem..

[91] Zhou X., Liu L., Zeng X. (2021). Research Progress on the Utilisation of Embedding Technology and Suitable Delivery Systems for Improving the Bioavailability of Nattokinase: A Review. Food Struct..

[92] Martínez-Ramos T., Benedito-Fort J., Watson N.J., Ruiz-López I.I., Che-Galicia G., Corona-Jiménez E. (2020). Effect of Solvent Composition and Its Interaction with Ultrasonic Energy on the Ultrasound-Assisted Extraction of Phenolic Compounds from Mango Peels (*Mangifera indica* L.). Food Bioprod. Process..

[93] Ouyang X., Zhou L., Xu X., Yang Z., Wang L., Lu L., Liu G., Zhang G. (2021). Preparation and Properties of Poly(MMA-Co-TMPTA)/Fragrance Microcapsules. Colloids Surf. A Physicochem. Eng. Asp..

[94] Jeoh T., Wong D.E., Strobel S.A., Hudnall K., Pereira N.R., Williams K.A., Arbaugh B.M., Cunniffe J.C., Scher H.B. (2021). How Alginate Properties Influence in Situ Internal Gelation in Crosslinked Alginate Microcapsules (CLAMs) Formed by Spray Drying. PLoS ONE.

[95] Hu Y., Liu S., Li X., Yuan T., Zou X., He Y., Dong X., Zhou W., Yang Z. (2018). Facile Preparation of Biocompatible Poly(l-Lactic Acid)-Modified Halloysite Nanotubes/Poly(ε-Caprolactone) Porous Scaffolds by Solvent Evaporation of Pickering Emulsion Templates. J. Mater. Sci..

[96] Yang X., Wang Y., Bai R., Ma H., Wang W., Sun H., Dong Y., Qu F., Tang Q., Guo T. (2019). Pickering Emulsion-Enhanced Interfacial Biocatalysis: Tailored Alginate Microparticles Act as Particulate Emulsifier and Enzyme Carrier. Green Chem..

[97] Yu L., Yang J.F., Guan B.Y., Lu Y., Lou X.W.D. (2018). Cover Picture: Hierarchical Hollow Nanoprisms Based on Ultrathin Ni-Fe Layered Double Hydroxide Nanosheets with Enhanced Electrocatalytic Activity towards Oxygen Evolution (Angew. Chem. Int. Ed. 1/2018). Angew. Chem. Int. Ed..

[98] Huang X.-N., Zhu J.-J., Xi Y.-K., Yin S.-W., Ngai T., Yang X.-Q. (2019). Protein-Based Pickering High Internal Phase Emulsions as Nutraceutical Vehicles of and the Template for Advanced Materials: A Perspective Paper. J. Agric. Food Chem..

[99] Li Y., Liu J., He X., Kong D., Zhou C., Wu H., Yang Z., Yang Z., Hu Y. (2020). Preparation of Cinnamon Oil-Loaded Antibacterial Composite Microcapsules by In Situ Polymerization of Pickering Emulsion Templates. Macromol. Mater. Eng..

[100] Wang C., Chen Y., Cui Y., Zhang T., Zhang D., Ma C., Chen S., Li H. (2021). Microencapsulation of Camellia Oil to Maintain Thermal and Oxidative Stability with Focus on Protective Mechanism. Int. J. Food Sci. Technol..

[101] Castejón N., Luna P., Señoráns F.J. (2021). Microencapsulation by Spray Drying of Omega-3 Lipids Extracted from Oilseeds and Microalgae: Effect on Polyunsaturated Fatty Acid Composition. LWT.

[102] Liu Y., Cao W., Wang J., Zhang L., Yang Y., Liu M., Wang H., Wang S. (2022). Preparation and Characterization of Perilla Essential Oil Composite Microcapsule Based on the Complex Coacervation and Interface Polymerization. J. Food Sci..

[103] Zhao Y., Liu R., Qi C., Li W., Rifky M., Zhang M., Xiao P., Wu T., Sui W. (2021). Mixing Oil-Based Microencapsulation of Garlic Essential Oil: Impact of Incorporating Three Commercial Vegetable Oils on the Stability of Emulsions. Foods.

[104] Kouamé K.J.E.-P., Bora A.F.M., Liu Y., Yu X., Sun Y., Hussain M., Md M., Coulibaly I., Li X., Liu L. (2023). Development and Characterization of Omega-3-Rich Flaxseed Oil Microcapsules and Evaluation of Its Stability and Release Behavior in Probiotic Millet Yogurt. Powder Technol..

[105] González A., Bordón M.G., Bustos M.C., Córdova Salazar K.L., Ribotta P.D., Martínez M.L. (2021). Study of the Incorporation of Native and Microencapsulated Chia Seed Oil on Pasta Properties. Int. J. Food Sci. Technol..

[106] Hakanen S.A., Damerau A., Ogrodowska D., Seubert A., Brandt W., Laaksonen O., Tańska M., Linderborg K.M. (2025). Sensory Profiles and Oxidative Stability of Linseed Oil Microencapsulated with Pea, Soy, and Whey Proteins in High-Fat Food Models. LWT.

[107] Zhang W., Chen Z., Yang R., Hua X., Zhao W., Guan S. (2021). Application of Caseinate Modified with Maillard Reaction for Improving Physicochemical Properties of High Load Flaxseed Oil Microcapsules. Eur. J. Lipid Sci. Technol..

[108] Siddiquy M., Al-Maqtari Q.A., Ghamry M., Othman N., Li J., Hlaing K.S.S., Zhang L. (2025). Microencapsulation Using a Novel Wall Material Prepared via Maillard Reaction-Derived Mung Bean Protein-Peach Gum Conjugates to Enhance Stability and Functionality of Chia Seed Oil. Int. J. Biol. Macromol..

[109] Copado C.N., Julio L.M., Diehl B.W.K., Ixtaina V.Y., Tomás M.C. (2021). Multilayer Microencapsulation of Chia Seed Oil by Spray-Drying Using Electrostatic Deposition Technology. LWT.

[110] Razavizadeh B.M., Shahidi Noghabi M., Molaveisi M. (2022). A Ternary Blending of Soy Protein Isolate/Maltodexterin/Inulin for Encapsulation Bioactive Oils: Optimization of Wall Material and Release Studies. Food Process. Preserv..

[111] Timilsena Y.P., Adhikari R., Barrow C.J., Adhikari B. (2016). Microencapsulation of Chia Seed Oil Using Chia Seed Protein Isolate chia Seed Gum Complex Coacervates. Int. J. Biol. Macromol..

[112] Zhu J., Li X., Liu L., Li Y., Qi B., Jiang L. (2022). Preparation of Spray-Dried Soybean Oil Body Microcapsules Using Maltodextrin: Effects of Dextrose Equivalence. LWT.

[113] Shisode P.S., Patil C.B., Mahulikar P.P. (2018). Preparation and Characterization of Microcapsules Containing Soybean Oil and Their Application in Self-Healing Anticorrosive Coatings. Polym.-Plast. Technol. Eng..

[114] Castel V., Rubiolo A.C., Carrara C.R. (2018). Brea Gum as Wall Material in the Microencapsulation of Corn Oil by Spray Drying: Effect of Inulin Addition. Food Res. Int..

[115] Le Priol L., Dagmey A., Morandat S., Saleh K., El Kirat K., Nesterenko A. (2019). Comparative Study of Plant Protein Extracts as Wall Materials for the Improvement of the Oxidative Stability of Sunflower Oil by Microencapsulation. Food Hydrocoll..

[116] Zhang H., Song G., Ma W., Guo M., Ling X., Yu D., Zhou W., Li L. (2023). Microencapsulation Protects the Biological Activity of Sea Buckthorn Seed Oil. Front. Nutr..

[117] Hoyos-Leyva J.D., Bello-Perez L.A., Agama-Acevedo J.E., Alvarez-Ramirez J., Jaramillo-Echeverry L.M. (2019). Characterization of Spray Drying Microencapsulation of Almond Oil into Taro Starch Spherical Aggregates. LWT.

[118] Zhang C., Zhou W., Xiang J., Chen H., Quek S.Y. (2022). Fabrication, Characterization, and Oxidative Stability of Perilla Seed Oil Emulsions and Microcapsules Stabilized by Protein and Polysaccharides. Food Process. Preserv..

[119] Glomm W.R., Molesworth P.P., Sandru E.M., Truong L.T., Brunsvik A., Johnsen H. (2021). Microencapsulation of Peppermint Oil by Complex Coacervation and Subsequent Spray Drying Using Bovine Serum Albumin/Gum Acacia and an Oxidized Starch Crosslinker. Appl. Sci..

[120] Javaid A., Imran A., Arshad M.U., Afzaal M., Asif Shah M. (2023). Formulation and Characterization of Micro-Emulsions of Peppermint and Coriander Oils Extracted by Using a Supercritical Fluid System. Int. J. Food Prop..

[121] Hernández-Fernández M.Á., García-Pinilla S., Ocampo-Salinas O.I., Gutiérrez-López G.F., Hernández-Sánchez H., Cornejo-Mazón M., Perea-Flores M.D.J., Dávila-Ortiz G. (2020). Microencapsulation of Vanilla Oleoresin (*V. planifolia* Andrews) by Complex. Coacervation and Spray. Drying: Physicochemical and Microstructural Characterization. Foods.

[122] Espinosa-Solís V., García-Tejeda Y.V., Portilla-Rivera O.M., Barrera-Figueroa V. (2021). Tailoring Olive Oil Microcapsules via Microfluidization of Pickering o/w Emulsions. Food Bioprocess. Technol..

[123] Başyiğit B., Yücetepe M., Karaaslan A., Karaaslan M. (2021). High Efficiency Microencapsulation of Extra Virgin Olive Oil (EVOO) with Novel Carrier Agents: Fruit Proteins. Mater. Today Commun..

[124] Devi N., Hazarika D., Deka C., Kakati D.K. (2012). Study of Complex Coacervation of Gelatin A and Sodium Alginate for Microencapsulation of Olive Oil. J. Macromol. Sci. Part A.

[125] Németh B., Németh Á.S., Ujhidy A., Tóth J., Trif L., Gyenis J., Feczkó T. (2018). Fully Bio-Originated Latent Heat Storing Calcium Alginate Microcapsules with High Coconut Oil Loading. Sol. Energy.

[126] Sapei L., Mustika P.C.B.W., Sutrisna P.D., Agustriyanto R., Setyopratomo P., Santoso G.V., Utama J.P., Indrawanto R. (2025). Inulin-Coated Virgin Coconut Oil (VCO) Powder Produced by Spray Drying. Appl. Food Res..

[127] Zhou D., Pan Y., Ye J., Jia J., Ma J., Ge F. (2017). Preparation of Walnut Oil Microcapsules Employing Soybean Protein Isolate and Maltodextrin with Enhanced Oxidation Stability of Walnut Oil. LWT-Food Sci. Technol..

[128] Lin D., Xiao L., Li S., Qin W., Loy D.A., Chen H., Zhang Q. (2022). Effects of Fructooligosaccharide and Soybean Protein Isolate in the Microencapsulation of Walnut Oil. Ind. Crops Prod..

[129] Kalkan F., Vanga S.K., Murugesan R., Orsat V., Raghavan V. (2017). Microencapsulation of Hazelnut Oil through Spray Drying. Dry. Technol..

[130] Guo Y., Bao Y., Chai Y. (2019). Preparation of Microcapsule Antioxidative Wall Materials of Pine Nut Oil by the Maillard Reaction. J. Sci. Food Agric..

[131] Bajac J., Nikolovski B., Lončarević I., Petrović J., Bajac B., Đurović S., Petrović L. (2022). Microencapsulation of Juniper Berry Essential Oil (*Juniperus communis* L.) by Spray Drying: Microcapsule Characterization and Release Kinetics of the Oil. Food Hydrocoll..

[132] Maji T.K., Hussain M.R. (2009). Microencapsulation of *Zanthoxylum limonella* Oil (ZLO) in Genipin Crosslinked Chitosan-Gelatin Complex for Mosquito Repellent Application. J. Appl. Polym. Sci..

[133] Gulzar S., Nilsuwan K., Raju N., Benjakul S. (2022). Whole Wheat Crackers Fortified with Mixed Shrimp Oil and Tea Seed Oil Microcapsules Prepared from Mung Bean Protein Isolate and Sodium Alginate. Foods.

[134] Yeh K.-W., Chang C.P., Yamamoto T., Dobashi T. (2011). Release Model of Alginate Microcapsules Containing Volatile Tea-Tree Oil. Colloids Surf. A Physicochem. Eng. Asp..

[135] Huang Q., Gong S., Han W., Chen Y., Shu X. (2020). Preparation of TTO/UF Resin Microcapsule via in Situ Polymerisation and Modelling of Its Slow Release. J. Microencapsul..

[136] Song F., Li Y., Wang B., Shen X., Wang H., Li R., Xia Q. (2022). Effect of Drying Method and Wall Material Composition on the Characteristics of Camellia Seed Oil Microcapsule Powder. J. Am. Oil Chem. Soc..

[137] Xu W., Sun H., Li H., Li Z., Zheng S., Luo D., Ning Y., Wang Y., Shah B.R. (2022). Preparation and Characterization of Tea Oil Powder with High Water Solubility Using Pickering Emulsion Template and Vacuum Freeze-Drying. LWT.

[138] Yang P., Du M., Cao L., Yu Z., Jiang S. (2020). Preparation and Characterization of Emulsion-Based Peony Seed Oil Microcapsule. J. Oleo Sci..

[139] Wang S., Shi Y., Han L. (2018). Development and Evaluation of Microencapsulated Peony Seed Oil Prepared by Spray Drying: Oxidative Stability and Its Release Behavior during in-Vitro Digestion. J. Food Eng..

[140] Charve J., Reineccius G.A. (2009). Encapsulation Performance of Proteins and Traditional Materials for Spray Dried Flavors. J. Agric. Food Chem..

[141] Shi Y., Wang S., Tu Z., Wang H., Li R., Zhang L., Huang T., Su T., Li C. (2016). Quality Evaluation of Peony Seed Oil Spray-Dried in Different Combinations of Wall Materials during Encapsulation and Storage. J. Food Sci. Technol..

[142] Lee K.-R., Kim E.-H., Kim K.-H., Park J.-S., Kim H.U. (2017). Vegetable Oil Production in Vegetative Plant Tissues. Plant Biotechnol. Rep..

[143] Razavizadeh B.M., Tabrizi P. (2021). Characterization of Fortified Compound Milk Chocolate with Microcapsulated Chia Seed Oil. LWT.

[144] Yu F., Li Z., Zhang T., Wei Y., Xue Y., Xue C. (2017). Influence of Encapsulation Techniques on the Structure, Physical Properties, and Thermal Stability of Fish Oil Microcapsules by Spray Drying. J. Food Process Eng..

[145] Kairam N., Kandi S., Sharma M. (2021). Effects on Bread and Oil Quality after Functionalization with Microencapsulated Chia Oil: Effects on Bread and Oil Quality after Functionalization with Microencapsulated Chia Oil. LWT.

[146] Wang Z., He Z., Zhang D., Chen X., Li H. (2022). Effect of Pepper (*Zanthoxylum bungeanum* Maxim.) Essential Oil on Quality Changes in Rabbit Meat Patty during Chilled Storage. J. Food Sci. Technol..

[147] Linke A., Hinrichs J., Kohlus R. (2020). Impact of the Powder Particle Size on the Oxidative Stability of Microencapsulated Oil. Powder Technol..

[148] Verkempinck S.H.E., Salvia-Trujillo L., Moens L.G., Charleer L., Van Loey A.M., Hendrickx M.E., Grauwet T. (2018). Emulsion Stability during Gastrointestinal Conditions Effects Lipid Digestion Kinetics. Food Chem..

[149] Hernández-Hernández E., Lira-Moreno C.Y., Guerrero-Legarreta I., Wild-Padua G., Di Pierro P., García-Almendárez B.E., Regalado-González C. (2017). Effect of Nanoemulsified and Microencapsulated Mexican Oregano (*Lippia graveolens* Kunth) Essential Oil Coatings on Quality of Fresh Pork Meat: Mexican Oregano Essential Oil Coatings on Quality of Fresh Pork Meat. J. Food Sci..

[150] Bolger Z., Brunton N.P., Monahan F.J. (2018). Impact of Inclusion of Flaxseed Oil (Pre-Emulsified or Encapsulated) on the Physical Characteristics of Chicken Sausages. J. Food Eng..

[151] de Conto L.C., Porto Oliveira R.S., Pereira Martin L.G., Chang Y.K., Steel C.J. (2012). Effects of the Addition of Microencapsulated Omega-3 and Rosemary Extract on the Technological and Sensory Quality of White Pan Bread. LWT-Food Sci. Technol..

[152] Cittadini A., Munekata P.E.S., Pateiro M., Sarriés M.V., Domínguez R., Lorenzo J.M. (2022). Microencapsulated Healthy Oil Mixtures to Enhance the Quality of Foal Pâtés. Foods.

[153] Bai Z., Yu R., Li J., Wang N., Wang Y., Niu L., Zhang Y. (2018). Application of Several Novel Natural Antioxidants to Inhibit Oxidation of Tree Peony Seed Oil. CyTA-J. Food.

[154] Locali-Pereira A.R., Lopes N.A., Menis-Henrique M.E.C., Janzantti N.S., Nicoletti V.R. (2020). Modulation of Volatile Release and Antimicrobial Properties of Pink Pepper Essential Oil by Microencapsulation in Single- and Double-Layer Structured Matrices. Int. J. Food Microbiol..

[155] Luna-Guevara J.J., Ochoa-Velasco C.E., Hernández-Carranza P., Guerrero-Beltrán J.A. (2017). Microencapsulation of Walnut, Peanut and Pecan Oils by Spray Drying. Food Struct..

